# Identification of peptide sequences as a measure of Anthrax vaccine stability during storage

**DOI:** 10.4161/hv.28443

**Published:** 2014-03-17

**Authors:** Gail Whiting, Jun X Wheeler, Sjoerd Rijpkema

**Affiliations:** 1 Division of Bacteriology; National Institute for Biological Standards and Control; Hertfordshire, UK; 2 Laboratory of Molecular Structure; National Institute for Biological Standards and Control; Hertfordshire, UK

**Keywords:** anthrax vaccine, peptides, protective antigen, mass spectrometry, stability, shelf life

## Abstract

The UK anthrax vaccine is an alum precipitate of a sterile filtrate of *Bacillus anthracis* Sterne culture (AVP). An increase in shelf life of AVP from 3 to 5 years prompted us to investigate the in vivo potency and the antigen content of 12 batches with a shelf life of 6.4 to 9.9 years and one bulk with a shelf life of 23.8 years. All batches, except for a 9.4-year-old batch, passed the potency test. Mass spectrometry (MS) and in-gel difference 2-dimensional gel electrophoresis (DIGE) were used to examine antigens of the pellet and supernatant of AVP. The pellet contained proteins with a MW in excess of 15 kDa. DIGE of desorbed proteins from the pellet revealed that with aging, 19 spots showed a significant change in size or intensity, a sign of protein degradation. MS identified 21 proteins including protective antigen (PA), enolase, lethal factor (LF), nucleoside diphosphate kinase, edema factor, and S-layer proteins. Fifteen proteins were detected for the first time including metabolic enzymes, iron binding proteins, and manganese dependent superoxide dismutase (MnSOD). The supernatant contained131 peptide sequences. Peptides representing septum formation inhibitor protein and repeat domain protein were most abundant. Five proteins were shared with the pellet: 2,3,4,5-tetrahydropyridine-6-dicarboxylate N-succinyltransferase, enolase, LF, MnSOD, and PA. The number of peptide sequences increased with age. Peptides from PA and LF appeared once batches exceeded their shelf life by 2 and 4 years, respectively. In conclusion, changes in antigen content resulting from decay or desorption only had a limited effect on in vivo potency of AVP. The presence of PA and LF peptides in the supernatant can inform on the age and stability of AVP.

## Introduction

The UK anthrax vaccine (AVP) is manufactured by Public Health England and has been in production since the 1950s. AVP was first described in 1965, and is an aluminum potassium sulfate (alum) -precipitated sterile culture filtrate of *Bacillus anthracis* Sterne strain.^1^^,^^2^ The main mediator of protection is the binding subunit of the anthrax toxin: protective antigen (PA). Besides PA, AVP contains many other antigens including enolase, lethal factor (LF), and edema factor (EF).^2^^-^^5^ PA is also the active agent in the Anthrax Vaccine Adsorbed (AVA) produced by Emergent Biosolutions^TM^, which is derived from the culture supernatant of non-virulent *B. anthracis* V770-NP1-R and uses aluminum hydroxide as adjuvant. By comparison this vaccine contains lower levels of LF and negligible levels of EF.^6^^,^^7^ Both EF and LF are enzymatic subunits that bind to multimeric PA to form the active toxins edema toxin (ET) and lethal toxin (LT) respectively. These toxins attack host cells and tissues in a variety of ways.^8^ Thus toxin neutralizing antibodies either directed to PA, LF, or EF can change the balance between pathogen and host to mitigate the outcome of anthrax and limit infection.

Due to their complex nature, the safety record of AVP and AVA has attracted the attention of the health care community. Following anthrax vaccination, the incidence of systemic side effects such as arthralgia, myalgia, and flu like syndrome was found to be significantly higher compared with immunization with vaccines based on purified antigens such as Tetanus Toxoid or Hepatitis A.^9^ However, studies among vaccinated British and American service personnel showed that side effects were mostly mild, localized at the site of injection and did not lead to an increase in medical consultations.^10^^-^^13^ Controversially, both anthrax vaccines were implicated in “Gulf War Syndrome” which was described in veterans of the first Gulf War.^14^^,^^15^ However a causal link with the anthrax vaccine remained unproven, either in experimental studies using animal models or in epidemiological studies among Gulf War veterans.^15^^-^^21^ Thus AVP and AVA are considered safe and efficacious for human use.

In a previous study, we analyzed the antigen content of AVP by 2-dimensional gel electrophoresis. We showed that the number of spots tended to decrease with age of the batch, indicating degradation of vaccine antigens. Mass spectrometry (MS) identified the presence of anthrax toxin subunits and several antigens, not associated with the toxin, such as enolase, nucleoside diphosphate kinase, heat shock proteins, and S-layer proteins.^5^ Whether antibodies to these components contribute to the protection and/or adverse effects in vaccinated individuals remains to be proven.

Recently, the shelf life of AVP was increased from 3 to 5 y. To increase our insight into the stability of the vaccine during storage, we examined the potency and antigen content of 15 final lots (batches) and one bulk by MS.

All batches met specifications at the time of release and the shelf life of the batches examined in this study ranged from 1 to 10 y. The vaccine bulk was not released for human use and was 23.8 y old when analyzed.

The potency of batches that had exceeded their shelf life was retested in the guinea pig challenge model, to ascertain the potency of the batch at the time of analysis.^22^

For analysis of the antigen content, batches of AVP were separated in a supernatant and a pellet fraction. The assumption was that the pellet contained proteins precipitated by the adjuvant and the supernatant contained dissociated or non-adsorbed protein fragments and oligopeptides. Supernatants were analyzed by reverse phase liquid chromatography (LC) followed by tandem peptide sequencing MS (LC-MS/MS). The proteins derived from desorbed pellets of individual batches were separated by in-gel difference 2-dimensional gel electrophoresis (2D-DIGE) and spots which showed a change in size or intensity, as the vaccine aged, were excised and submitted to MS/MS for analysis.

Here we confirm and extend our previous analysis of protein components of AVP. Due to improved sensitivity of LC-MS/MS and the combination of MS/MS and 2D-DIGE, novel proteins and peptides were identified in the supernatant and the pellet respectively. We show that antigens precipitated by the adjuvant notably enolase, EF, LF, and PA, are subject to degradation during prolonged storage and that peptides are abundant in the supernatant. The number of peptide sequences in the supernatant is related to the age of the batch and PA and LF peptides were only detected in the supernatant of batches, which had exceeded their shelf life. These findings are discussed in relation to vaccine potency to determine their relevance as markers for efficacy and stability of AVP.

## Results

### Potency of vaccine batches

All AVP batches, except for batch 1, were intended for human use and met the release specifications for AVP as described in the European Pharmacopeia monograph 2188.^22^ The potency of 10 batches which had exceeded their shelf life was retested. The results are given in Table 1. Only batch 3 was considered to have failed the potency test.

**Table:** **Table 1.** Characteristics of AVP batches and fractions thereof used in this study

Batch ID	Age in years^1^ Our	Outcome of potency test P/F^2^		Supernatants analyzed by LC/MS-MS					Pellets analyzed by 2D-DIGE and LC/MS-MS				
			No. of SHD used	Protein content in µg					No. of SHD used	Protein content in µg		Analyzed in 2D-DIGE	2D-DIGE spots analyzed by LC/MS-MS
				C4 eluate	C8 eluate	C18 eluate	Combined eluates	per SHD		Combined pellets	Per SHD		
*1 ^3^*	23.6	–	12	63.9	17.8	11.0	92.7	7.7	–	–	–	–	–
2	9.9	0.7 (0.4–1.1)P	18	100.5	43.8	25.7	169.9	9.4	3	50.4	16.8	Yes	Yes
3	9.4	0.5 (0.3–0.8)F	18	71.2	253.0	26.5	350.7	19.5	3	48.9	16.3	Yes	Yes
4	8.8	1.1 (0.6–2.0)P	9	75.8	20.6	9.2	105.6	11.7	–	–	–	–	–
5	8.7	–	19	67.0	79.5	26.5	173.0	9.1	3	66.0	22.0	Yes	Yes
6	8.7	1.1 (0.7–1.7)P	8	55.3	19.5	8.9	83.7	10.5	–	–	–	–	–
7	8.0	0.9 (0.4–1.7)P	9	73.9	19.7	8.0	101.6	11.3	3	45.0	15.0	Yes	Yes
8	7.8	1.0 (0.4–2.3)P	8	202.8	18.5	9.5	230.8	28.9	–	–	–	–	–
9	7.8	1.0 (0.5–2.0)P	9	56.5	20.9	11.2	88.7	9.9	–	–	–	–	–
10	7.8	–	19	84.1	61.6	27.3	173.1	9.1	3	49.2	16.4	Yes	Yes
11	7.3	1.0 (0.6–1.8)P	19	108.2	74.4	28.3	210.9	11.1	3	42.6	14.2	Yes	Yes
12	7.2	0.8 (0.5–1.5) P	9	57.4	19.9	12.4	89.8	10.0	3	42.3	14.1	Yes	Yes
13	6.4	0.8 (0.4–1.5)P	9	27.0	56.9	15.2	99.1	11.0	3	53.4	17.8	–^4^	–
14	1.1	1.6 (0.9–2.9)P^5^	–	–	–	–	–	–	5	75.0	15.0	Yes^6^	–
15	1.0	1.1 (0.6–2.2)P^5^	13	354.7	27.8	20.5	403.0	31.0	5	79.5	15.9	Yes^6^	–
16	1.0	1.5 (0.8–3.2)P^5^	5	105.9	7.4	4.4	117.7	23.5	5	82.5	16.5	Yes^6^	–

^1^Age calculated at the time of testing. ^2^Potency of the batch relative to reference freeze-dried AVP standard NIBSC 99/790, (95% confidence interval) *P* = pass, F = Fail. ^3^Bulk 1 was used as a reference standard for the rabbit edema test and was not released as a final lot. ^4^Poor 2D-DIGE result excluded due to lack of material. ^5^Tested at release to market. ^6^2D-DIGE spot pattern analyzed only. –, not done.

### Analysis of vaccine supernatant by LC- MS/MS

Supernatant of multiple SHDs of 14 batches and one bulk were used to prepare sequential solid phase extraction (SPE) eluates for analysis by LC-MS/MS. The protein content of SPE eluates from batches which had exceeded their shelf life and bulk 1 was variable (Table 1). No correlation between protein content and age of the material was observed for this group. However, the average protein content of SPE eluates from batches which had exceeded their shelf life (n = 13, 12.2 µg ± 5.7) was significantly lower (*P* = 0.017) compared with the average protein content (n = 2, 27.3 µg ± 5.3) of SPE eluates from batches that were within shelf life. Sodium dodecyl polyacrylamide gel electrophoresis (SDS-PAGE) analysis revealed that proteins or protein fragments were absent from the supernatant with the exception of the supernatants of bulk 1, which contained a 20 kD fragment (results not shown).

Following trypsin digestion, LC-MS/MS identified 212 peptides and 207 of these matched the *B. anthracis* database and are thus members of the *B. anthracis* proteome. Of these, 131 peptide sequences were unique, 91 peptides were a product of trypsin digestion and 40 peptides were the result of other protease activity or spontaneous degradation (marked *, see Table 2; Supplementary Data 1). The 212 peptides identified represented 98 proteins, including 14 hypothetical proteins (Supplementary Data 1). Table 2 summarizes the sequences of the peptides that were present in the supernatant of 3 batches or more and that corresponds to 12 proteins, including 2 hypothetical proteins.

**Table:** **Table 2.** Most frequently identified peptide sequences in vaccine supernatant and the *B. anthracis* protein they represent

*B. anthracis* protein/ NCBI accession number	Present in supernatant of batch	Peptide sequence (position)
Septum formation inhibitor /NP_846895	3, 4, 5, 6, 7, 8, 9, 10, 11, 13, 15, 16	MEEKKQQNVTIK (1–12)
Repeat domain protein/ EJY91466	1, 3, 5, 6, 8, 9, 11, 12, 13, 15, 16	RLFLSSTEGDADLIGDQALFG*(230–250)
Protective antigen/AAA22637	1, 2, 3, 4, 5, 6, 7, 9, 10, 11, 12	GPTVPDRDNDGIPDS* (172–186)
2,3,4,5-Tetrahydropyridine-2, 6-dicarboxylate N- succinyltransferase /NP_846430	2, 3, 4, 5, 6, 9, 12	AGVIEPPSAKP* (155–165)
Amino acid permease/ YP_028011	6, 7, 8, 9, 10, 11, 16	STELGEQKLNKTK (16–28)
Hypothetical protein/ NP_843342	6, 8, 9, 10, 11, 13, 15	ELLSENIR (127–134)
HAD family hydrolase, subfamily II*B.* /NP_844477	1, 4, 5, 10, 12,	SQFNALGINTI* (51–61)
Hypothetical protein/ZP_00391958	3, 4, 6, 10, 13	IEEPENEDKLENK (293–305)
Enolase /NP_847538	1, 3, 6, 9	IELDGTPNKGKLG*(95–107)
Alcohol dehydrogenase/NP_844655	3, 6, 9	IKVSDIKPGQP* (156–166)
Inosine-uridine preferring nucleoside hydrolase family protein/NP_845876	10, 12, 13	YGNVTQEKATSNAAYLLQLAG*(36–56)
Lethal factor/CAC93932	1, 2, 3	IQVDSSNPLSEK (365–376)

*Indicates peptide not cleaved by trypsin.

The number of peptides identified in the supernatant increased with vaccine age and this relationship was especially pronounced when the bulk (batch 1) was included in the analysis (Fig. 1A and B). The supernatant of batch 1 contained 44 peptides derived from 35 proteins, the largest number of proteins identified for any of the supernatants. The same trend was observed for PA peptides (Fig. 1A and B). The most frequently identified amino acid sequences were “MEEKKQQNVTIK” representing the septum formation inhibitor (SFI) protein in 12 batches and ‘RLFLSSTEGDADLIGDQALFG’ representing the repeat domain (RD) protein found in 11 batches (Table 2; Supplementary Data 1). The age of the batch did not correlate with the presence of peptides derived from the RD protein or the SFI protein.

**Figure:**
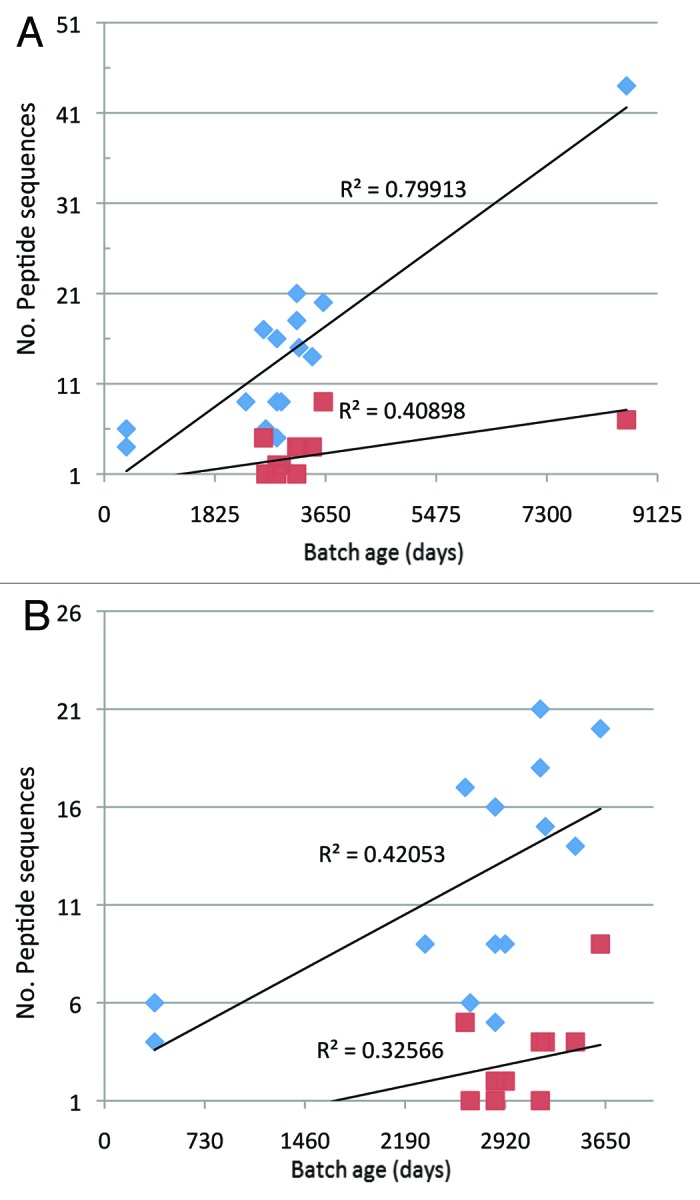
**Figure 1.** The abundance of peptide sequences in the eluate derived from PA as well as all antigens is associated with age of the anthrax vaccine batch. (**A**) all batches; (**B**) batches excluding sample 1; (◆) all peptides and (■) PA peptides.

Nineteen different PA peptides were found in the supernatant of 11 batches with an age of 7 y or more. PA peptide sequences GPTVPDRDNDGIPDS* and DNLQLPELK occurred most frequently as part of 15 longer amino acid sequences found in 9 batches and 10 sequences found in 7 batches respectively. The majority of the peptides (74%) in the vaccine supernatant were derived from 4 amino acid sequences of Domain 1. Most of these contained the core sequences mentioned above (Table 3). Three peptide sequences (16%) were positioned in Domains 2–4 (Table 3; Supplementary Data 1). Peptides representing the enzyme 2,3,4,5-tetrahydropyridine-2, 6-dicarboxylate N-succinyltransferase (THP succinyltransferase) were present in the supernatant of 7 batches with a shelf life in excess of 7 y.

**Table:** **Table 3.** Protective antigen peptides detected in the vaccine supernatant and pellet

PA Domain^1^	Oligo peptide^1,2^	Position	Identified in
1	EVKQENRLLNESESSSQG*	1–18	Supernatant
1	SDEYTFATSADNHVTmWVDDQEVINK	74–99	Supernatant/Pellet
1	K**EVISSDNLQLPELK**	153–167	Supernatant/Pellet
1	LYWTDSQNK	144–152	Pellet
1	R**STSAGPTVPDRDNDGIPDSLE**VEGYTVDVK	167–197	Supernatant/Pellet
1	RTFLSPWISNIHEK	210–223	Pellet
1	SSPEK**WSTASDPYSDFEK**	221–238	Pellet
1	IDKNVSPEAR	243–252	Pellet
1–2	**HPLVAAYPIVHVDMENIILSK**NEDQSTQNTDSQTR	253–287	Pellet
2	TWAETmGLNTADTAR	345–359	Pellet
2	YVNTGTAPIYNVLPTTSLVLGK	366–388	Pellet
2	AK**ENQLSQILAPNNYYPSK**	396–414	Pellet
2	NLAPIALNAQDDFSSTPITmNYNQFLELEK	415–444	Pellet
2	LDTDQVYGNIATYNFENGR	450–468	Pellet
2–3	VR**VDTGSNWSEVLPQIQETTAR**	469–490	Supernatant/Pellet
3	DLNLVER	497–503	Pellet
3	R**IAAVNPSDPLETTKPDmTL**K	504–525	Pellet
3	IAFGFNEPNGNLQYQGK	529–545	Pellet
3	NQLAELNATNIYTVLDK	564–580	Pellet
3	LNAKmNILIR	583–592	Pellet
4	FHYDR**NNIAVGADESVVK**	596–613	Supernatant/Pellet
4	EVINSSTEGLLLNIDK	617–633	Pellet
4	IVEIEDTEGLKE*	642–654	Supernatant
4	YDmLNISSLR	660–669	Pellet
4	QDGK**TFIDFKK**	670–680	Pellet
4	YNDK**LPLYISNPNYK**	681–695	Pellet
4	VNVYAVTK	696–703	Pellet
4	ENTIINPSENGDTSTNGIK	704–722	Pellet

^1^Protective antigen protein sequence AAA22637. ^2^The longest oligo peptide sequence is given. Sequences in bold were detected individually (Supplementary Data 2). m, oxidized form of methionine, possibly a site of post translational modification. *Indicates peptide not cleaved by trypsin.

The presence of LF peptides was limited to 2 batches with an age of more than 9 y and bulk 1 (see Table 2).

EF peptides were not detected in the supernatant for any of the batches tested. Peptide sequences representing 12 proteins were found in 2 batches, 74 peptide sequences occurred in one batch only. Five peptide sequences could not be identified by the search of the *B. anthracis* proteome (Supplementary Data 1). The latter group may represent carry over proteins from media components or contaminants.

### Analysis of vaccine pellets by 2D-DIGE

The protein content from pellets of 11 batches was determined following dissolution of the pellet (Table 1). The average protein content of the pellet from one SHD did not differ significantly for batches 2, 3, 5, 7, and 10–13, which were over 6 y of age (n = 8, 16.6 ± 2.6 µg) compared with batches 14–16 that were within shelf life (n = 3, 15.8 ± 0.8 µg). The desalting required for 2D-DIGE analysis may have reduced the protein yield and therefore our results may underestimate the total amount of protein absorbed to the alum adjuvant, these results can be used to compare the amount of protein in the pellet of each batch.

2D-DIGE analysis of the protein content of batches 14–16 revealed an average of 1103 ± 99 spots per gel and 595 of the larger well delineated spots were matched across gel images generated from all gels. The vast majority of these spots (91.9%) did not change significantly in abundance and only 8.1% of the spots showed a significant change (*P* ≤ 0.05) in protein abundance between the batches.

2D-DIGE analysis of 7 batches with a shelf life of 7 y or older showed an average of 919 ± 81 protein spots per gel. Batch 13 was excluded from this analysis due to low protein content of the pellet (Table 1). A total of 504 well-delineated spots were matched across 33 gel images generated from 11 gels. Of these, 248 spots (49%) showed a significant change (*P* ≤ 0.05) in protein abundance. Many of these changes were related to the age of the batch (Table 4). The most pronounced changes in spot size and intensity were found when patterns from batch 2 (9.9 y) were compared with batch 12 (7.2 y).

**Table:** **Table 4.** 2D-DIGE spots that showed a change in protein level

Spot No.	Observed change	*P* value
1	Decrease with age of batch	0.0012
2	Decrease with age of batch	0.0029
3	Decrease with age of batch	0.0039
4	Increase with age of batch	0.00012
5	Increase with age of batch	0.0011
6	Decrease with age of batch	0.0089
7	Decrease with age of batch	0.0015
8	Decrease with age of batch	0.00036
9	Decrease with age of batch	0.033
10	Decrease with age of batch	0.011
11	Decrease with age of batch	0.073^1^
12	Decrease with age of batch	0.02
13	Decrease with age of batch	0.0021
14	Decrease only in batch 5	0.00002
15	Decrease with age of batch	0.00027
16	Decrease with age of batch	0.00031
17	Decrease only in batch 5	0.0045
18	Increase with age of batch	0.000015
19	Decrease only in batch 5	0.00029
20	Decrease only in batch 5	0.018
21	Decrease only in batch 5	0.0014
22	Decrease only in batch 5	0.000018
23	Increase with age of batch	0.0045
24	Decrease with age of batch	0.002
25	Decrease with age of batch	0.00084

^1^Change is not significant due to large size of spot.

### Identification of protein species in excised spots

Twenty-four spots which showed a significant change in protein abundance and one spot which showed a decrease in size were excised (Fig. 2 and Table 4). Indicating that proteins present in these spots are susceptible to degradation during prolonged storage of the vaccine over time. Spots 14, 17, 19, 20, 21, and 22 only showed a change for batch 5 (Table 4). Proteins were extracted for MS/MS analysis and peptide sequences were identified in 19 of the 25 spots, representing 21 proteins (Supplementary Data 2). The remainder of the spots did not contain sufficient protein. PA peptides that were detected in the pellet are given in Table 3. The identity of all extracted proteins is given in Table 5. Of the 379 peptide sequences, 159 peptides were unique, and all were the result of trypsin digestion (Supplementary Data 2). The analysis confirms that PA is the major protein in the pellet fraction. PA was detected in 15 spots (79%) and represented by 146 peptide sequences (39% of total) of which 36 peptide sequences are unique (Table 3; Supplementary Data 2). The location of the PA peptides detected in the pellet fraction differed when compared with PA peptides of the supernatant. In the pellet fraction, 12 peptides belonged to Domain 1 (33%), followed by 11 peptides (31%) from Domain 4, eight peptides (22%) from Domain 2 and five peptides from 3 (14%; see Table 3; Supplementary Data 1 and 2). Thus compared with the supernatant, domain 1 peptides of PA were under represented in the pellet fraction.

**Figure:**
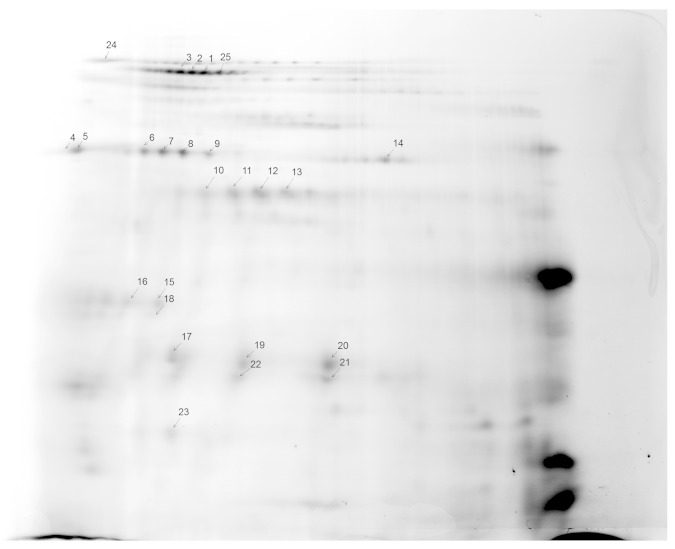
**Figure 2.** Position of the excised spots on the 2D-DIGE gel stained with Novex colloidal blue. The spot number is related to numbers presented in Tables 4 and 5.

**Table:** **Table 5.** Proteins identified by MS/MS in 19 spots excised from 2D-DIGE gel

Spot No.	*B. anthracis* protein	NCBI accession number	*Mr*/p*I*	No. of peptides (coverage)
1	Protective antigen	AAA22637	82.8/5.90	18 (34%)
	Lethal factor	CAC93932	93.7/5.95	18 (24.1%)
3	Protective antigen fragment	AAA22637	82.6/ 5.72	10 (15.8%)
	Lethal factor endopeptidase	CAC93932	93.7/5.95	6 (6.9%)
4	Enolase	NP_847538	46.4/4.77	25 (62.4%)
	Protective antigen fragment	AAA22637	82.6/5.72	2 (4.5%)
5	Enolase	NP_847538	46.4/4.77	23 (58.9%)
	Protective antigen fragment	AAA22637	82.6/5.72	2 (3.8%)
6	Protective antigen	AAA22637	82.8/5.90	20 (34.0%)
	Enolase	NP_847538	46.4/4.77	14 (47.1%)
	Lethal factor	CAC93932	93.7/5.95	8 (12.5%)
	Glucose-6-phosphate isomerase	NP_847316	50.3/5.14	4 (16.4%)
	Serine hydroxymethyltransferase	NP_847716	45.1/5.91	2 (5.3%)
7	Protective antigen	AAA22637	82.8/5.90	20 (31.1%)
	Enolase	NP_847538	46.4/4.77	10 (35.5%)
	Lethal factor	CAC93932	93.7/5.95	3 (3.7%)
	Glucose-6-phosphate isomerase	NP_847316	50.3/5.14	2 (4.4%)
8	Protective antigen fragment	AAA22637	62.8/5.83	12 (21.9%)
9	Protective antigen fragment	AAA22637	62.8/5.83	21 (45.1%)
11	Protective antigen fragment	AAA22637	26.6/7.46	2 (18.9%)
	Lipoprotein, Bmp family	NP_846172	38.3/8.48	2 (6.5%)
	6-Phosphofructokinase	NP_847047	34.3/6.29	2 (8.5%)
12	Protective antigen fragment	AAA22637	26.6/7.46	3 (23.2%)
	Iron compound ABC transporter, iron compound-binding protein	NP_847506	35.9/6.71	6 (21%)
	Lipoprotein, Bmp family	NP_846172	38.3/8.48	2 (6.5%)
13	Protective antigen fragment	AAA22637	82.6/5.72	11 (16.5%)
	Iron compound ABC transporter, iron compound-binding protein	NP_847506	35.9/6.71	3 (11.7%)
	Lethal factor	CAC93932	93.7/5.95	2 (2.7%)
	6-Phosphofructokinase	NP_847047	34.3/6.29	3 (9.7%)
	Lipoprotein, Bmp family	NP_846172	38.3/8.48	2 (6.5%)
	Edema factor fragment	AAA79215	29.4/5.19	2 (5.2%)
14	Lethal factor	CAC93932	93.7/5.95	5 (12.5%)
16	Protective antigen fragment	AAA22637	82.6/5.72	11 (2.1%)
	Lethal factor	CAC93932	93.7/5.95	6 (8.2%)
	DNA-binding response regulator	NP_845926	25.8/5.17	4 (12.9%)
	Formate acetyltransferase	NP_843045	84.5/5.96	4 (6.1%)
	6-Phosphofructokinase	NP_847047	34.3/6.29	3 (11.3%)
	S-layer protein	NP_052786	76.2/9.16	3 (4.3%)
	Sporulation-control protein Spo0M	NP_844693	28.8/4.94	4 (13.5%)
	Transaldolase	NP_845714	24.0/5.47	3 (20.3%)
	Purine nucleoside phosphorylase DeoD-type	NP_843936	25.7/5.19	2 (9.4%)
	2,3,4,5-Tetrahydropyridine-2,6-dicarboxylate N-succinyltransferase	NP_846430	25.7/5.54	2 (10.4%)
18	Manganese dependent superoxide dismutase fragment	NP_846724	15.2/5.41	2 (21.1%)
	S-layer protein	NP_052786	76.2/9.16	2 (2.9%)
19	Protective antigen fragment	AAA22637	26.6/7.46	2 (6.4%)
20	Protective antigen fragment	AAA22637	26.6/7.46	3 (10.3%)
	Hypothetical protein	NP_843713	19.9/7.31	2 (6.4%)
	S-layer protein	NP_052786	76.2/9.16	3 (3.7%)
	Glucose-6-phosphate isomerase	NP_847316	50.3/5.14	2 (6.2%)
	Lethal factor fragment	CAC93932	30.3/5.14	2 (7.6%)
23	Nucleoside diphosphate kinase	NP_843987	16.5/5.55	5 (39.9%)
24	Iron transport-associated protein	YP_006211623	98.9/5.62	23 (26.1%)
25	Protective antigen	AAA22637	82.8/5.90	10 (18.9%)
	Lethal factor	CAC93932	93.7/5.95	12 (14.8%)
	S-layer protein SAP	NP_843397	86.5/7.30	2 (12.6%)
	Edema factor	AAA79215	88.8/6.89	2 (3.7%)

Other abundant proteins were LF, present in 9 spots (47%; 54 peptide sequences of which 21 are unique), enolase present in 4 spots (69 peptide sequences of which 21 are unique) and S-layer protein present in spots 16, 18, 20, and 25 (10 peptide sequences of which 6 are unique; see Table 5; Supplementary Data 2). The presence of these proteins and nucleoside diphosphate kinase and the edema factor is in line with findings of our previous study.^5^ Full size LF and PA tended to co-migrate (see Table 5, spots 1, 3, 6, 7, 16, and 25). PA was also present as lower MW fragments (see Table 5, spots 11, 12, 19, and 20).

This study also identified 14 proteins for the first time in AVP—6-phosphofructokinase, DNA-binding response regulator, formate acetyltransferase, glucose-6-phosphate isomerase, 2 iron binding proteins, lipoprotein Bmp family, manganese dependent superoxide dismutase (MnSOD), phosphoesterase, purine nucleoside phosphorylase DeoD-type, serine hydroxymethyltransferase, sporulation-control protein Spo0M, THP succinyltransferase, and transaldolase (Table 5, Supplementary Data 2).

Peptides sequences of 5 precipitated proteins were identified in the supernatant of AVP—enolase, LF, MnSOD, PA, and THP succinyltransferase. Peptide sequences of SFI and RD proteins which were abundant in the supernatants were not detected in the pellet fraction. We attribute the detection of a wider range of antigens to improved extraction procedures and an increased sensitivity of the DIGE technique and the MS/MS.

## Discussion

AVP is a complex mixture of PA, LF, EF, and numerous other antigens. Proteins are precipitated by the adjuvant and reside in the pellet, whereas peptides are found in the supernatant. We analyzed the antigen content of the pellet and the potency of AVP batches which had exceeded their shelf life. All but one of the AVP batches passed the in vivo potency test, including batches which exceeded their shelf life by a considerable margin (Table 1). Batches that were within shelf life were not examined. Despite this omission, the evidence that AVP is efficacious up to and beyond its shelf life is compelling.

Analysis of the pellet fraction indicated that protein decay is common among batches which had expired. However, the lack information on the decay of specific antigens during shelf life, limits our understanding of the significance of this process for vaccine stability and the effect on the antigens identified in Table 5. We did examine the peptide composition of the vaccine supernatant in 3 1-y-old batches and this provided a useful reference for the results of batches, which exceeded their shelf life (Table 2).

The presence of PA, EF, enolase, LF, and S-layer proteins in the vaccine pellet confirmed findings of our previous study.^5^ Two members of the S-layer protein family were identified, *sap* located on the chromosome and one S-layer protein located on pXO1.^23^^,^^24^ Both enolase and S-layer proteins have been detected in spent medium of *B. anthracis* cultures, which explains their abundance in the pellet of AV, and both proteins are also secreted by *B. anthracis* under culture conditions that simulate the host environment.^25^^,^^26^ Thus besides the toxin subunits, enolase and S-layer proteins may elicit antibodies following vaccination. Enolase binds human plasminogen and is considered a virulence factor that could be involved in tissue lysis.^27^ S-layer proteins are immunogenic and S-layer vaccines protected mice against anthrax challenge.^28^^-^^31^

Also present in AVP are—enzymes required for vegetative growth such as transaldolase and THP succinyltransferase, membrane proteins such as iron transport proteins, lipoprotein Bmp, and MnSOD (Table 5). Like enolase and S-layer proteins, these antigens reflect the use of culture supernatant as the source of AVP. THP succinyltransferase is required for the biosynthesis of Diaminopimelic acid, a substrate for Sortase C and essential for anchoring of surface proteins to the cell envelope of *B. anthracis*.^32^^,^^33^ The iron transport-associated protein (Table 5, spot 24) is part of a family of iron-regulated surface determinant proteins containing NEAr Transporter domains.^34^ These proteins are secreted during culture and were shown to be immunogenic in convalescing guinea pigs and rabbits and in humans following immunisation with AVA.^34^^,^^35^ Antibodies to transaldolase and enolase were found in recipients of AVA.^35^ Thus several of the proteins identified in the pellet of AVP are immunogenic and shared with AVA. MnSOD, is present in the exosporium of *B. anthracis* and is considered a virulence factor which protects the bacterium against reactive oxygen species in the host and during culture.^36^

The proteins mentioned above were all detected in spots that with one exception showed a reduction in size or intensity for older AVP batches, indicating loss of these proteins from the pellet with increased age of AVP. Indeed, a considerable proportion of spots decreased in size among batches that were over 6 y of age compared with spot patterns of 1-y-old batches. In addition the total number of spots per individual batch was lower in batches that had exceeded their shelf life. Previously we noted a reduction in the number of spots for older batches of AVP and an increase in the inter-batch variation with increasing age of the samples.^5^ Thus both studies point to a decay of protein antigens during prolonged storage of AVP.

Peptides derived from a total of 98 proteins were detected in the supernatant and only 5 proteins were detected in the pellet: PA, enolase, LF, MnSOD, and THP succinyltransferase (Tables 2 and 5). The supernatants of 1-y-old batches contained 4 or 6 peptide sequences and as the age of the batch increased, the number of peptide sequences steadily increased to 44 for the 23.8 y old bulk. Peptides from the pellet fraction all resulted from trypsin digestion, which is part of the sample preparation. Whereas a large proportion of the peptides in the supernatant resulted from other protease activity, for example during fermentation or during temperature controlled storage of the vaccine. Although data are lacking for 2 to 5 y old batches, there is a correlation between age and the number of peptide sequences and this correlation was also found for PA peptides. However, it remains unclear whether protein decay during storage is a gradual process or whether it accelerates as batches age beyond their shelf life.

Despite the presence of peptides from SFI and RD proteins in the supernatant of 75% of the batches, SFI and RD proteins were not detected in the pellet. The function of these proteins is unknown. The RD protein was recently identified in the genome of *B. anthracis* isolated from cattle, but a function was not allocated.^37^ One protein, likely to be related to RD, was shown to be associated with the spore capsule in *Bacillus* strains.^38^ The septum plays a role in the onset of sporulation.^39^ This implies that SFI protein may therefore have a role during the vegetative growth of the Sterne strain although currently its function is unknown.

Most of the PA peptides in the supernatant belonged to domain 1 and are represented by sequences “GPTVPDRDNDGIPDS” and “DNLQLPELK” (Table 3). In the pellet fraction, PA peptides from domains 2–4 dominated. This observation is of particular interest for vaccine efficacy and stability. Domain 1 is the N-terminal domain of PA, which contains the furin cleavage site ^164^RKKR^167^. Furin is a host protease that removes a 20 kD N-terminal fragment from domain 1.^40^ Upon activation by furin cleavage, PA assembles into a heptamer ring-shaped structure with a large hydrophobic surface, which is then able to bind EF and LF. Domain 2 and domain 3 are required for the polypeptide translocation and oligomerization respectively. Domain 4 is involved in the binding of the PA complex to the host cell.^40^^,^^41^ Peptide GPTVPDRDNDGIPDS was not the result of trypsin digestion suggesting that specific *Bacillus* proteases may have cleaved this peptide during fermentation or that this peptide fragment may have dissociated from the adjuvant spontaneously during storage. Accelerated degradation of an experimental rPA vaccine, which contained Alum hydroxide as adjuvant, showed that domain 1 epitopes became more accessible for binding to monoclonal antibodies (Mabs) compared with epitopes in domains 2 to 4.^42^ These changes were explained as a result of the unfolding of parts of PA, which exposes normally hidden epitopes which are situated at domain interfaces and the disruption of conformational epitopes at the surface of PA.^42^ Thus domain 1 of PA is likely to be more unstable than other parts of the molecule and therefore may be susceptible to desorption. This hypothesis supports our finding that domain 1 is prone to dissociation and degradation during storage. A study in mice showed that adsorption of rPA to alum adjuvant did not induce higher titers of toxin neutralizing antibodies.^43^ However, unfolding of the PA molecule did affect the induction of toxin neutralizing antibodies in vaccinated mice.^42^ Toxin neutralizing antibodies interfere with the formation of PA heptamers, the formation of the toxin complex and/or prevent binding of anthrax toxin to the host receptor.^44^ In humans, anthrax vaccination induces an anti-PA IgG response which is mainly directed toward domain 1, but only a minority of these antibodies are able to neutralize LT, which contrasts with the higher proportion of toxin neutralizing antibodies generated that bind to PA_63_.^45^^,^^46^ Human or humanised Mabs that bind to domain 1, specifically the furin cleavage site, and 2 mouse Mabs which bind close to the furin cleavage site are protective in vitro and in a mouse model.^47^^-^^50^

Epitope VKNKRTFLSPWISNIHEK of domain 1 is recognized by toxin neutralizing murine Mab 19D9 and present in adsorbed PA_83 _(Table 3; Supplementary Data).^48^ Steric hindrance reduces enzyme activity and slows or prevents cleavage of PA_83_, which in turn would limit formation of active LT in vivo. However, the efficacy of anti-domain 1 antibodies could be affected by the propensity of domain 1 to be freely available in the serum of infected individuals. Free subunits of domain 1 are then able to bind to anti-domain 1 antibodies, rendering these antibodies incapable of neutralizing the holo- toxin.^46^^,^^48^ In view of these observations, epitopes of domain 1 may be considered to be of less importance for the induction of toxin neutralizing antibodies than epitopes of domains 3 and 4. Indeed most therapeutic Mabs, including the recently licensed Raxibacumab^TM^, are directed against domains 3 or 4.^44^^,^^51^

The amount of PA is not specified for release of AVP.^22^ The amount of adsorbed PA was not determined in these batches and thus it remains unclear whether the presence of PA peptides in the supernatant of AVP is associated with low levels of adsorbed PA and loss of potency. For monovalent rPA vaccines it would be of interest to determine if the presence of PA peptides in the supernatant could act as a marker for the stability, for example following accelerated degradation. In particular, because stability of the rPA vaccine was problematic during the initial manufacture of this vaccine.^52^

Despite the abundance of LF in the pellet, LF peptides were only detected in the supernatant of bulk 1 and 9 to 10 y old production batches. The presence of both PA and LF peptides and the failure of batch 3 to pass the potency test may be associated. However, we cannot rule out that this effect is due to a batch specific variation and therefore further work is required to support this observation.

The absence of EF peptides in the supernatant and the limited presence of EF among precipitated proteins is in line with previous observations that fermentation conditions for production of AVP result in high levels of PA followed by LF and that levels of EF are very low.^2^^,^^4^^,^^5^

In conclusion, MS analysis of the supernatant of adsorbed vaccines like AVP, can provide valuable information about vaccine stability and antigen content. With continuing advances in this area, such methods should become more accessible to national control laboratories. However, as the current study shows the selection of relevant markers for efficacy and stability is not straight forward for a complex vaccine such as AVP. In vivo assays remain a crucial component of the batch release process to ascertain the potency and safety of AVP.

## Materials and Methods

### Anthrax vaccine batches

Fifteen final lots of AVP and one bulk used in this study are given in Table 1. All were donated by Public Health England Porton (previously Health Protection Agency). In this study, the oldest sample, the bulk, was assigned number 1 and the final lots (batches) were assigned numbers 2 to 16. The bulk was not released for use in humans. All batches met specifications and were released. Batches 2–13 and bulk 1 had a shelf life that exceeded 5 y at the time of testing. Batches 14–16 were all in date at the time of testing (Table 1).

### Guinea pig potency test

The potency of the AVP batches was measured as the relative potency compared with freeze-dried AVP reference standard NIBSC 99/790. The potency test used in this study was a modification of the challenge assay in guinea pigs described in Monograph 2188 of the European Pharmacopeia.^22^ The modifications related to the dilutions of the vaccine used to immunise guinea pigs and the pass criteria. Groups of animals received 2 immunizations of AVP diluted 1/10, 1/30, 1/90, or 1/270 SHD. To reflect the achievable precision of the assay, the pass criteria applied to batches, which had exceeded their shelf life were: the relative potency exceeds 1.0 or the 95% confidence interval includes 1.0 and the lower 95% confidence limit is not less than 33% of the relative potency.

### Protein detection

The amount of protein in the vaccine supernatant or pellet was determined by BCA (Thermo Scientific Pierce) or Bradford assay (Bio-Rad).

### Direct peptide sequencing of vaccine supernatant by LC-MS/MS

Excipients were removed from vaccine supernatants by SPE prior to LC-MS/MS analysis. SPE used 3 types of reverse phase matrixes represented by cartridges C4, C8, and C18 (CHROMABOND® HR-Xpert polymers, Macherey Nagel). Cartridge C4 retains the hydrophobic peptides, C18 retains smaller less hydrophobic peptides and C8 is an intermediary in both respects. Cartridges were pre-treated with 3 mL of 50% acetonitrile (ACN), 0.1% trifluoroacetic acid (TFA) and equilibrated with 3 mL of 0.1% TFA. The supernatants of several SHDs were pooled (Table 1) and passed through a cartridge 3 times followed by a wash step (3 mL of 0.1% TFA). Following passage through a C4 cartridge, the non-binding material was passed through a C8 cartridge and subsequently through a C18 cartridge. Bound peptides were eluted with 1.5 mL of 50% ACN and 0.1% TFA. Eluates from C4, C8, and C18 cartridges were dried and reconstituted in 100 µL of 50 mM ammonium bicarbonate pH 8.5.^53^

The amount of protein in a 50 µL aliquot of the eluate from each sample was measured by BCA assay. Trypsin digestion was performed on the remaining sample unless the sample contained more than 20 μg of protein in which case a volume equivalent to 20 μg of protein was used. The sample was incubated in the presence of 1% Rapigest (Waters; sodium 3-[{2-methyl-2-undecyl-1,3-dioxolan-4-yl}methoxy]-1-propanesulfonate) at 100 °C for 5 min to solubilise proteins and peptides. Trypsin was added once the sample was at RT (ratio of 20:1 w/w enzyme/protein) and incubated at 37 °C overnight. Hydrochloric acid was added to terminate digestion and ensure breakdown of Rapigest.

The digests were analyzed using LC-MS/MS equipped with a nano-electrospray ion source and 2 mass detectors, linear trap and orbitrap, coupled with an Ultimate 3000 nano-LC system, comprising a solvent degasser, a loading pump, a nano-pump, and a thermostatted autosampler (Thermo Fisher). After automated injection, the extracted peptides were trapped in a cartridge (PepMap reversed phase C18; 5 µm [100 Å], 300 µm internal diameter × 5 mm length) and eluted on to a C18 reversed phase nano-column (3 µm [100 Å], 75 µm internal diameter × 15 cm length), and followed by a 60 min separation under a column flow rate of 0.3 µL/min using linear gradient from 5–70% acetonitrile and 0.1% formic acid. After a first survey MS scan (from m/z 400–2000) in the linear trap, the 5 most intense ions were sequentially isolated and passed to the orbitrap for accurate mass measurement with the resolution of 30 000. These were then fragmented in the linear ion trap at collision induced energy of 35%. The total cycle time was approximately 30 ms. Data was collected in data dependent MS/MS mode with dynamic exclusion set to 2 counts.

Mass spectra were processed and peptides were identified by a search of the Proteome Discoverer *v* 1.2 with built-in Sequest (Thermo Electron) against *B. anthracis* FASTA database using either trypsin as the enzyme or no enzyme for specific or non-specific searches to identify both trypsinized peptides as well as peptides generated by *Bacillus* proteases or by natural decay during storage at 2–8 °C. For the search, initial mass tolerances for protein identification by MS were set to 10 ppm and the peptide must be in rank 1 to be considered as a positive identification. Subsequently, the identity of the protein and the location of the oligopeptide sequences (Supplementary Data 1) were confirmed by searching against the non-redundant *B. anthracis* proteins in the Protein Blast database of the US. National Centre for Biotechnology Information (NCBI).^54^

### Preparation of anthrax vaccine pellet for 2D-DIGE analysis

Antigens from pellets of selected batches (Table 1) were prepared as follows. For batches 14–16, a homogenous suspension of 2.5 mL (5 SHDs) was concentrated using Amicon Ultra 3K 0.5 mL centrifugal filter units (Millipore) to yield 0.5 mL concentrated vaccine. Anthrax antigens were desorbed from the alum by the addition of 15 µL 10M NaOH to 0.5 mL concentrated vaccine and the sample was vortexed until the suspension cleared. The solution was immediately neutralized by the addition of 15 µL of 3M sodium citrate and centrifuged briefly to remove insoluble material. Vaccine antigens were precipitated using 2D clean up kit (GE Healthcare) and stored at −20 °C until required.

Due to limited availability of batches 2, 3, 5, 7, 10, 11, 12, and 13, antigens were prepared from 1.5 mL (3 SHDs) vaccine. The samples were centrifuged for 5 min at 13 000 g. The pellet was resuspended in 100 µL sterile distilled water; proteins were desorbed and precipitated as described above.

### Labeling of anthrax vaccine proteins with fluorescent dye

Proteins from desorbed pellets of AVP were resuspended in 50 µL of sample buffer (30 mM Tris, 7 M Urea, 2 M Thiourea, 4% (w/v) 3-[{3-cholamidopropyl} Dimethylammonio]-1-propanesulfonate, pH 8.5 [CHAPS]). The pH was determined by spotting a small volume (~1 µL) onto a pH indicator strip. If necessary the pH was adjusted to 8.5. The protein concentration was determined and the samples were stored in sample buffer at -70 °C until required. Two CyDye DIGE Fluor minimal dyes (NHS-Cy3 and Cy5) were used to label proteins derived from batches 14, 15, and 16. The internal standard, consisting of equal amounts of protein from each vaccine batch included in the experiment, was prepared by pooling the vaccine protein preparations. The internal standard was labeled with 400 pmol Cy3 per 50 μg of protein. Proteins, from the desorbed pellet of 3 batches of AVP were labeled with 400 pmol Cy5 per 50 µg protein.

Three CyDye DIGE Fluor minimal dyes (NHS-Cy2, Cy3, and Cy5) were used to label proteins derived from batches 2, 3, 5, 7, 10, 11, and 12. The internal standard, consisting of equal amounts of protein from each batch included in the experiment, was prepared by pooling the vaccine protein preparations. The internal standard was labeled with 400 pmol Cy2 per 50 μg of protein. Proteins, from desorbed pellets of a batch of AVP were labeled with 400 pmol Cy3 or Cy5 per 50 µg protein. Protein labeling was performed according to the manufacturer’s instructions (GE Healthcare). Briefly, samples were mixed with the appropriate concentration of dye and incubated on ice in the dark for 30 min. The reaction was terminated by the addition of lysine (10 pmol per 400 pmol dye) followed by a further 10 min incubation on ice in the dark.

### Isoelectric focusing and SDS-PAGE

Isoelectric focusing (IEF) and SDS-PAGE were performed according to the manufacturer’s recommendations. For IEF of batches 14–16, 2 different protein samples were run on a single 2D gel, the Cy3-labeled pooled internal control and a Cy5-labeled AVP test sample. For IEF of batches 2, 3, 5, 7, 10, 11, and 12, three different protein samples were run on a single 2D gel, the Cy2-labeled pooled internal control and a Cy3 and a Cy5-labeled AVP test sample. The differently labeled protein samples were combined in a single microfuge tube and mixed. The labeled protein samples were diluted in rehydration buffer (7 M Urea, 2 M Thiourea, 2% [w/v] CHAPS, 2% [w/v] IPG Buffer pH 3–11, 18 mM Dithiothreitol [DTT], trace of Bromophenol blue) to the required final volume of 600 µL. Samples were loaded onto Immobiline DryStrips (pH 3–11 NL, 24 cm, GE Healthcare) for 16 h using the in-gel rehydration technique. Isoelectric focusing was performed for 100 kVh using the Ettan IPGphor 3 isoelectric focusing system (GE Healthcare). Prior to SDS-PAGE separation, the IPG strips were equilibrated for 10 min in equilibration buffer (6 M urea, 75 mM Tris, pH 8.8, 30% v/v glycerol, 2% w/v SDS) containing 130 mM DTT and for a further 10 min in equilibration buffer containing 150 mM Iodoacetamide. The strips were placed on top of a 12% polyacrylamide gel (24 cm × 24 cm × 1 mm) cast between low fluorescence glass plates using Next Gen 2DEoptimizer gel caster and then sealed with 0.5% agarose in SDS electrophoresis running buffer(25 mM Tris, 192 mM Glycine, 0.2% [w/v] SDS). Gels were run on the ETTAN 6 vertical gel system (GE Healthcare).

### Image acquisition, analysis, and spot picking

2D-DIGE gels were scanned within the low fluorescence glass plates. Imaging was performed at 10 µm resolution using a Typhoon 9410 variable mode imager (GE Healthcare). Cy2, Cy3, and Cy5 scans were made of each gel as required. The appropriate excitation and emission wavelengths were used for each dye—Cy2 with a blue 488 nm laser and a 520 nm emission filter; Cy3 with a green 532 nm laser and a 580 emission filter; and Cy5 with a red 633 nm laser and a 670 nm emission filter.

A prescan was performed to determine the optimal photomultiplier tube voltage required for the scanning of each image. 2D-DIGE analysis of 1-y-old batches 14, 15, and 16 comprised 4 replicates of each batch. Twelve gels were analyzed with each gel containing a Cy3-labeled internal standard and a Cy5-labeled test sample. 2D-DIGE analysis of batches 2, 3, 5, 7, 10, 11, and 12, which were over 7 y of age comprised 11 gels. Each gel contained a Cy2-labeled internal standard and a Cy3 and Cy5-labeled vaccine sample. At least 3 replicates of each vaccine batch were included in the analysis. Image analysis was performed using the biological variance analysis module of the DeCyder differential analysis software (GE Healthcare, *v* 7.2). Quantitative comparison of spots across multiple gels was enabled by the incorporation of an internal standard in the experimental design. Spot patterns from the different gels were matched using the internal standard present on each gel to allow comparison and statistical analysis of spot-volume ratios. Spot detection, background subtraction, and normalization were performed, however manual editing was required to merge and separate spots and identify landmark spots before automatic matching could occur. For spot picking, selected gels were stained overnight with Novex colloidal blue stain and then destained briefly in deionised water (Fig. 2). Protein spots of interest were excised from gels.^53^

### Identification of peptides in 2-D DIGE spots

Peptides were extracted from 2D DIGE spots as described by Wheeler et al.^53^ In brief, excised 2-D gel spots were washed with ammonium bicarbonate, then 50% ACN in ammonium bicarbonate and finally in 100% ACN. Each step lasted for 30 min. This procedure was repeated once. Fifty µL of ammonium bicarbonate containing 0.1 µg Trypsin was added to the dehydrated gel spot and incubated o/n at RT. Peptides were extracted sequentially in 4 steps: adding 50 µL of 1% TFA, transferring the supernatant, then adding 100 µL of 50% ACN in 0.2% TFA twice and transferring supernatant twice and finally 100 µL of 100% ACN was added. The supernatants were pooled and dried in a centrifugal evaporator. The pellet was reconstituted in 100 µL 0.1% formic acid and subjected to LC-MS/MS analysis. Mass spectra were processed and extracted peptides were identified by a search of the Proteome Discoverer *v* 1.2 with built-in Sequest (Thermo Electron) against *B. anthracis* FASTA database using trypsin as the enzyme. Peptide sequences (Supplementary Data 2) were confirmed by searching against the non-redundant *B. anthracis* proteins in the Protein Blast database of NCBI.^54^

### Statistical analysis

One way ANOVA was used to analyze the protein content of vaccine fractions and to determine the significance of variations in spot intensity for different batches following 2D-DIGE analysis.

## Supplementary Material

Additional material

## Abbreviations:

ACN: acetonitrile
CHAPs: 3-[(3-cholamidopropyl) dimethylammonio]-1-propanesulfonate
AVA: anthrax vaccine adsorbed
AVP: anthrax vaccine, alum precipitated sterile filtrate
2D-DIGE: in-gel difference 2 dimensional gel electrophoresis
DTT: dithiothreitol
EF: edema factor
ET: edema toxin
kD: kilo Dalton
kVh: kilo Volt hours
LC: liquid chromatography
LF: lethal factor
LT: lethal toxin
Mab: monoclonal antibody
MS: mass spectrometry
MW: molecular weight
PA: protective antigen
RD: repeat domain
SDS-PAGE: sodium dodecyl polyacrylamide gel electrophoresis
SFI: septum formation inhibitor
SHD: standard human dose
SOD: superoxide dismutase
SPE: sequential solid phase extraction
TFA: trifluoroacetic acid
THP succinyltransferase: 2,3,4,5-tetrahydropyridine-2, 6-dicarboxylate N-succinyltransferase

## Notes

10.4161/hv.28443
