# Supplementary material for: Identification of peptide sequences as a measure of Anthrax vaccine stability during storage
Source: Hum Vaccin Immunother. 2014 Mar 17;10(6):1669–81. doi: 10.4161/hv.28443 (PMC4185962; doi:10.4161/hv.28443)
Supplement: Additional material [file hvi-10-1669-s01.pdf]

Landes Bioscience
www.landesbioscience.com

Supplemental Material to:

Gail Whiting, Jun X Wheeler, and Sjoerd Rijpkema

Identi cation of peptide sequences as a measure of Anthrax vaccine stability during storage

Human Vaccines & Immunotherapeutics 2014; 10(6)
http://dx.doi.org/10.4161/hv.28443

www.landesbioscience.com/journals/vaccines/article/28443

<u>SUPPLEMENTARY DATA 1</u>

Identification of peptides in anthrax vaccine supernatant

Vaccine batch	*B. anthracis* protein/NCBI accession number	Peptides sequenced by LC-MS/MS	AA Position
**1**	Protective antigen/AAA22637	EVISSDNLQLPELK<br>VISSDNLQLPELK<br>TSAGPTVPDRDNDGIPDS*<br>TSAGPTVPDRDNDGIPDSLE*<br>GPTVPDRDNDGIPDSLE*<br>SEVLPQIQETTAR<br>NNIAVGADESVVK	D1 144-157<br>D1 145-157<br>D1 169-196<br>D1 169-198<br>D1 172-196<br>D2/3 478-490<br>D4 601-613
	Lethal factor/CAC93932	DSLSEEEKELLNR<br>IQVDSSNPLSEK	352-364<br>365-376
	Phosphoribosylamine-glycine ligase /NP_842849	FAQSEKVEKVYVAPGNEGMR<br>FGDPETEVVLPR	19-38<br>288-299
	O-acetylserine /NP_846820	NGGKAG*	210-215
	Acyl carrier protein/NP_846230	VEETEVVPAASFK	18-30
	Adenylosuccinate lyase/NP_842840	EDVKKIR	34-40
	Aminopeptidase/NP_847758	QVENAVSK	169-176
	Carboxyl-terminal protease /ZP_05186175	LVEGAIQGMLSTLK	74-87
	Cell wall surface anchor family protein /NP_847261	EAAQVQPK	89-96
	Cell wall anchor domain-containing protein/NP_845653	QENIQIPAAQVNDAISKTSEK	217-237
	Dlp-2 Chain A	TNVVEVLNK	4-12
	Cold shock protein Csp*B.* /NP_845864	TLEEGQEVTFEVEQGNR	40-56
	Enolase/NP_847538	AVGDEGGFAPNLGSNR	201-216
	Ferredoxin/NP_843955	VADESFDGDALKFE*	69-82
	Glycine cleavage system protein H /NP_847408	VELSDASQVEK	103-113
	HAD family hydrolase/NP_846519	DERAICWLA*	24-32
	Manganese dependent superoxide dismutase/ NP_846724	SVEELVANLNEVPEAIR	54-70
	NADH dehydrogenase subunit N /NP_847690	ITGSTNIVDMQKV*	186-198
	Phage-related minor tail protein/ZP_00393935	GVEDLHGATSKIDASPAVKFQK	96-117
	Prophage LambdaBa02, major capsid protein/NP_846330	GGTLSQEAR	119-127
	Repeat domain protein/ EJY91466	RLFLSSTEGDADLIGDQALFG*	230-250
	Respiratory nitrate reductase, alpha subunit/YP_028240	GDQFVADRFLNASDIGRETK	350-369
	30S ribosomal protein/NP_847869	IIRPGVEEEAQK	9-20
	50S ribosomal protein/NP_842668	LEEVGAAVEVK	109-119

*: Indicates peptide not cleaved by trypsin.

SUPPLEMENTARY DATA 1 ctd.

Identification of peptides in anthrax vaccine supernatant

Vaccine batch	***B. anthracis*** **protein/NCBI accession number**	**Peptides sequenced by LC-MS/MS**	**AA Position**
**1 ctd**	Ribosomal subunit interface protein /NP_847591	GENIEVTPALK	7-17
	Sensor histidine kinase/NP_844605	ESAGIYEK	109-116
	SpoII SA like protein/ZP_00392739	TWYGLFIVSVTVYFMIKGIDLTLWK	40-64
	Thioredoxin/NP_846964	VVKVDVDENQETAR	52-65
	Triose phosphate isomerise/NP_847540	TFDLVAGQVTK	139-149
	Hypothetical protein/NP_847643	AEANTDSEILGTLK	96-109
	Hypothetical protein/YP_006208132	KALEESGLSFK	85-95
	Hypothetical protein/ZP_05184708	DAMNKNVFVSADEKMKWLL*	567-585
	Hypothetical protein/NP_843315	AQEAAEAQAAAK	222-233
	Not characterised	VDYENGSAISSSILAEYNVKR<br>LDKWYYK	
**2**	Protective antigen/AAA22637	VDDQEVINK<br>EVISSDNLQLPELK<br>DNLQLPELK<br>GPTVPDRDNDGIPDSLE*<br>GPTVPDRDNDGIPDS*<br>PTVPDRDNDGIPDS*<br>VEGYTVDVK<br>IEDTEGLKE*<br>EDTEGLKE*	D1 91-99<br>D1 144-157<br>D1 149-157<br>D1 172-188<br>D1 172-186<br>D1 173-186<br>D1 189-197<br>D4 646-654<br>D4 647-654
	Acyltransferase /NP_844056	YEELDSIR	4-11
	6-aminohexanoate-dimer hydrolase /NP_845632	NNGEYNGKQI*	316-325
	Lethal factor/CAC93932	IQVDSSNPLSEK	365-376
	Chaperonin GroEL/ZP_00390655	TGGEVITEELGR	294-305
	Cold shock protein CspB/NP_845864	VEGGEDVFVHF*	20-30
	DNA gyrase subunit A/AAP43927	IAELVRDKK	278-286
	Glucose-1-phosphate adenylyltransferase /NP_847308	MPGAK	332-336
	Oligopeptide ABC transporter, substrate binding protein/NP_845910	KSTTTSNNGGEEK	34-46
	Phosphoglycerate mutase /ZP_05184256	LSEAREAGAI*	35-42
	Spore maturation protein/NP_843944	IAEEAGLLKK	57-66
	2,3,4,5-Tetrahydropyridine-2, 6-dicarboxylate N-succinyltransferase /NP_846430	AGVIEPPSAKP*	155-165

*: Indicates peptide not cleaved by trypsin.

SUPPLEMENTARY DATA 1 ctd.

Identification of peptides in anthrax vaccine supernatant

Vaccine batch	*B. anthracis* protein/NCBI accession number	Peptides sequenced by LC-MS/MS	AA Position
**3**	Protective antigen/AAA22637	EVKQENRLLNESESSSQG*<br>DNLQLPELKQK<br>GPTVPDRDNDGIPDSLE*<br>IVEIEDTEGLKE*	D1 1-18<br>D1 149-159<br>D1 172-188<br>D4 643-654
	Acyl-CoA dehydrogenase /YP_028630	VPKENILGDVNK	220-231
	Alcohol dehydrogenase/NP_844655	IKVSDIKPGQP*	156-166
	Lethal factor/CAC93932	DLVDSTDNTKINR	458-470
	Enolase /NP_847538	DIAVATNAGQIKTG*	379-392
	2-methylcitrate dehydratase /NP_844732	VREVLEMMIKAHEIQGVIALENSLNR	139-164
	Repeat domain protein/ EJY91466	RLFLSSTEGDADLIGDQALFG*	230-250
	Septum formation inhibitor /NP_846895	MEEKKQQNVTIK	1-12
	2,3,4,5-Tetrahydropyridine-2, 6-dicarboxylate N-succinyltransferase /NP_846430	AGVIEPPSAK	155-164
	Hypothetical protein/ZP_00391958	IEEPENEDKLENK	293-305
	Hypothetical protein/NP_847479	LLVVGGAGSLFVDEEKTTRVFETPGFPK	96-123
**4**	Protective antigen/AAA22637	EVISSDNLQLPELK<br>DNLQLPELK<br>GPTVPDRDNDGIPDS*<br>GPTVPDRDNDGIPDSLE*	D1 144-157<br>D1 149-157<br>D1 172-186<br>D1 172-188
	ATP synthase β subunit/NP_847705	ELQDIIAILGMDELSEEDKLVVHRAR	377-402
	Deoxynucleoside kinase/NP_842585	GRPMEQQTPIEYWK	155-168
	Glucose-1-phosphate adenylyl transferase /NP_847308	MPGAK	332-336
	HAD family hydrolase, subfamily II*B.* /NP_844477	SQFNALGINTI*	51-61
	immune inhibitor A metallo protease /NP_843199	DYDKIKRGVGVPTYIDQSVTKSNRPGV*	448-474
	Kinase associated protein *B.* /NP_847327	AFREQTNIPE*	66-75
	Repeat domain protein/ EJY91466	TETAVNTIISTPTK	184-197
	Septum formation inhibitor /NP_846895	MEEKKQQNVTIK	1-12
	2,3,4,5-Tetrahydropyridine-2, 6-dicarboxylate N-succinyltransferase /NP_846430	AGVIEPPSAKP*	155-165
	Hypothetical protein/ZP_00391958	IEEPENE*	293-300
	Hypothetical protein/NP_652833	VLYAEGAMHKEK	28-39

*: Indicates peptide not cleaved by trypsin.

SUPPLEMENTARY DATA 1 ctd.

Identification of peptides in anthrax vaccine supernatant

Vaccine batch	*B. anthracis* protein/NCBI accession number	Peptides sequenced by LC-MS/MS	AA Position
**5**	ABC transporter, ATP-binding protein/NP_845918	KMSKGMK	127-133
	ATP synthase β subunit/NP_847705	ELQDIIAILGMDELSEEDKLVVHRAR	377-402
	60 kDa chaperonin/NP_842820	TGGEVITEELGR	294-305
	DadA family oxidoreductase /NP_845069	VGAISLHIDEKKLDQMEER	89-107
	Deoxynucleoside kinase/NP_842585	GRPMEQQTPIEYWK	155-168
	HAD family hydrolase, subfamily II*B.* /NP_844477	SQFNALGINTI*	51-61
	3-Hydroxyacyl-CoA dehydrogenase /NP_847744	VQDLKQEQLDR	31-41
	Protective antigen/AAA22637	GPTVPDRDNDGIPDSLE*	D1 172-188
	Recombinase A /NP_846162	LGVNIDELLLSQPDTGEQGLEIAEALVR	105-132
	PTS system, trehalose specific IIBC component /NP_843162	EATVADVK	87-94
	Pyruvate kinase /NP_847046	TKVLNSGTVKNK	142-153
	Repeat domain protein/ EJY91466	RLFLSSTEGDADLIGDQALFG*	230-250
	50S Ribosomal protein L5 /ZP_05203220	LVSVSLPR	103-110
	Sensor histidine kinase/NP_844605	ESAGIYEK	109-116
	Septum formation inhibitor /NP_846895	MEEKKQQNVTIK	1-12
	2,3,4,5-Tetrahydropyridine-2, 6-dicarboxylate N-succinyltransferase /NP_846430	AGVIEPPSAKP*	155-165
	Transcriptional regulator, LysR family /NP_845819	VDLAFILDECK	141-151
	Transcriptional regulator, Xre family /ZP_15957990	ECLQMIATIR	133-142

*: Indicates peptide not cleaved by trypsin.

SUPPLEMENTARY DATA 1 ctd.

Identification of peptides in anthrax vaccine supernatant

Vaccine batch	*B. anthracis* protein/NCBI accession number	Peptides sequenced by LC-MS/MS	AA Position
**6**	Protective antigen/AAA22637	EVKQENRLLNESESSSQG*<br>VDDQEVINKA*<br>VDDQENINKASN*<br>GPTVPDRDNDGIPDS*	D1 1-18<br>D1 91-100<br>D1 91-102<br>D1 172-186
	ABC transporter, ATP-binding /ZP_02213439	EGKLVATGTTEELK	175-188
	Alcohol dehydrogenase/NP_844655	IKVSDIKPGQP*	156-166
	Amino acid permease/YP_028011	STELGEQKLNKTK	16-28
	Bifunctional phosphoribosyl aminoimidazole carboxamide formyltransferase /NP_842848	DESTISIPTKREPSE*	382-396
	Cold shock DNA binding protein/ZP_05204931	AIQGEGFKTL*	32-41
	Enolase /NP_847538	IELDGTPNKGKLG*	95-107
	Malate dehydrogenase /YP_021492	QVDPNEVAEKTK	384-394
	Methanol dehydrogenase regulatory protein; magnesium chelatase /ZP_05182734	FTPDLLPSDVTGVSI*	72-86
	1-pyrroline-5-carboxylate dehydrogenase/YP_026575	KHEPFTDF*	6-13
	Repeat domain protein/ EJY91466	RLFLSSTEGDADLIGDQALFG*	230-250
	Ribonuclease R/NP_847510	DFKEAKVLVDEE*	423-434
	Septum formation inhibitor /NP_846895	MEEKKQQNVTIK	1-12
	2,3,4,5-Tetrahydropyridine-2, 6-dicarboxylate N-succinyltransferase /NP_846430	AGVIEPPSAKP*	155-165
	Hypothetical protein/NP_843342	ELLSENIR	127-134
	Hypothetical protein/ZP_05150161	EENKELNKLSK	12-22
	Hypothetical protein/ZP_00391958	IEEPENEDKLENK	293-305
	Hypothetical protein/NP_845432	FEAELEEARTAR	93-104

*: Indicates peptide not cleaved by trypsin.

SUPPLEMENTARY DATA 1 ctd.

Identification of peptides in anthrax vaccine supernatant

Vaccine batch	*B. anthracis* protein/NCBI accession number	Peptides sequenced by LC-MS/MS	AA Position
**7**	Protective antigen/AAA22637	EVISSDNLQLPELK<br>GPTVPDRDNDGIPDS*	D1 144-157<br>D1 172-186
	Amino acid permease/YP_028011	STELGEQKLNKTK	16-28
	Imidazolonepropionase	QNIGGTTCVIKTAG*	
	Recombinase A /NP_846162	LGVNIDELLLSQPDTGEQGLEIAEALVR	105-132
	Rod shape determining protein MreD /NP_846896	LLSTLLLNAIFLFIVCFPLRRYLVR	144-168
	Septum formation inhibitor /NP_846895	MEEKKQQNVTIK	1-12
	Tryptophan synthase α subunit /NP_843726	DEIHSYLEKVKSHTHLPVVAGFGISTK	188-214
	Hypothetical protein/NP_844758	SELESGNIPVEK	102-113
**8**	Amino acid permease/YP_028011	STELGEQKLNKTK	16-28
	Repeat domain protein/ EJY91466	RLFLSSTEGDADLIGDQALFG*	230-250
	Glycosyltransferase, group 2 family protein/NP_843696	LGDCYAKLGLWEKAVESCVK	239-258
	Septum formation inhibitor /NP_846895	MEEKKQQNVTIK	1-12
	Hypothetical protein/NP_843342	ELLSENIR	127-134

*: Indicates peptide not cleaved by trypsin.

SUPPLEMENTARY DATA 1 ctd.

Identification of peptides in anthrax vaccine supernatant

Vaccine batch	*B. anthracis* protein/NCBI accession number	Peptides sequenced by LC-MS/MS	AA Position
**9**	Enolase /NP_847538	IELDGTPNKGKLG*<br>DLAGEGRTQTS*	95-107<br>257-264
	Acyl-CoA dehydrogenase /YP_028630	VPKENILGDVNK	220-231
	Alcohol dehydrogenase/NP_844655	IKVSDIKPGQP*	156-166
	Amino acid permease/ YP_028011	STELGEQKLNKTK	16-28
	LPXTG-motif cell wall anchor domain containing protein/ZP_02399493	SQGGNGSQGGNVKDNAKKQ*	223-241
	Protective antigen/AAA22637	DNLQLPELKQK	D1 149-159
	Putative lipoprotein/ZP_05185287	LSDAQGSGTVIGR	148-160
	1-pyrroline-5-carboxylate dehydrogenase/YP_026575	KHEPFTDF*	6-13
	Repeat domain protein/ EJY91466	RLFLSSTEGDADLIGDQALFG*	230-250
	Septum formation inhibitor /NP_846895	MEEKKQQNVTIK	1-12
	2,3,4,5-Tetrahydropyridine-2, 6-dicarboxylate N-succinyltransferase /NP_846430	AGVIEPPSAKP*	155-165
	tRNA pseudouridine synthase A /YP_026742	HDFTAFSNAKSKKKSMVRE*	152-170
	Hypothetical protein/ NP_843342	ELLSENIR	127-134
	Not characterised	DMTEGKSLFIETK<br>SKEENMTENRK	
**10**	Protective antigen/AAA22637	GPTVPDRDNDGIPDS*<br>GPTVPDRDNDGIPDSLE*	D1 172-186<br>D1 172-188
	Amino acid permease/ YP_028011	STELGEQKLNKTK	16-28
	Glycosyltransferase, group 2 family protein/NP_843696	LGDCYAKLGLWEKAVESCVK	239-258
	HAD family hydrolase, subfamily IIB /NP_844477	VMQALSETUNLEK	119-131
	Inosine-uridine preferring nucleoside hydrolase family protein/NP_845876	YGNVTQEKATSNAAYLLQLAG *	36-56
	Septum formation inhibitor /NP_846895	MEEKKQQNVTIK	1-12
	Hypothetical protein/ NP_843342	ELLSENIR	127-134
	Hypothetical protein/ZP_00391958	IEEPENEDKLENK	293-305

*: Indicates peptide not cleaved by trypsin.

SUPPLEMENTARY DATA 1 ctd.

Identification of peptides in anthrax vaccine supernatant

Vaccine batch	*B. anthracis* protein/NCBI accession number	Peptides sequenced by LC-MS/MS	AA Position
**11**	Amino acid permease/ YP_028011	STELGEQKLNKTK	16-28
	Protective antigen/AAA22637	EVISSDNLQLPELK	D1 173-186
	Repeat domain protein/ EJY91466	RLFLSSTEGDADLIGDQALFG*	230-250
	Septum formation inhibitor /NP_846895	MEEKKQQNVTIK	1-12
	UDP diphospho-muramoyl pentapeptide α-N-acetyl glucosaminyl transferase / NP_846702	AGSNAIFEFLTLQKPMLLIPLSKFASR	260-286
	Hypothetical protein/NP_843342	ELLSENIR	127-134
**12**	Protective antigen/AAA22637	VDDQEVINK<br>GPTVPDRDNDGIPDSLE*<br>GPTVPDRDNDGIPDS*<br>IVEIEDTEGLKE*<br>IEDTEGLKE*	D1 91-99<br>D1 172-186<br>D1 172-188<br>D4 643-654<br>D4 645-654
	Chaperone protein Hsp40/BAK52478	IPAGIDNGQQIR	166-177
	DNA binding protein/NP_846949	GLSIPLSR	66-73
	DNA topoisomerase/NP_844316	TEGLGTEATR	512-521
	HAD family hydrolase, subfamily II*B.* /NP_844477	SQFNALGINTI*	51-61
	Indolepyruvate decarboxylase /NP_844861	ETLDIKPFISESLSMTEEFNPKAHMV*	344-369
	Inosine-uridine preferring nucleoside hydrolase family protein/NP_845876	YGNVTQEKATSNAAYLLQLAG *	36-56
	IS10R transposase/ ZP_02399961	LMVLRASVALHGR	107-119
	Repeat domain protein/ EJY91466	RLFLSSTEGDADLIGDQALFG*	230-250
	2,3,4,5-Tetrahydropyridine-2, 6-dicarboxylate N-succinyltransferase /NP_846430	AGVIEPPSAKP*	155-165
	Transcriptional regulator, Xre family /ZP_15957990	ECLQMIATIR	133-142
	Hypothetical protein/NP_844933	IAILGANGK	7-15
	Hypothetical protein /NP_847688	MESMIASLEKNDAK	34-47

*: Indicates peptide not cleaved by trypsin.

<u>SUPPLEMENTARY DATA 1 ctd.</u>

Identification of peptides in anthrax vaccine supernatant

Vaccine batch	*B. anthracis* protein/NCBI accession number	Peptides sequenced by LC-MS/MS	AA Position
**13**	BNR repeat containing protein /YP_027894	RLFLCMER	3-10
	Repeat domain protein/ EJY91466	RLFLSSTEGDADLIGDQALFG*	230-250
	Heat shock protein 60 /BAB83630	VGNDGVITLEESK	77-89
	Inosine-uridine preferring nucleoside hydrolase family protein/NP_845876	YGNVTQEKATSNAAYLLQLAG*	36-56
	Oligopeptide ABC transporter substrate-binding protein/NP_843417	FVLAESFTPSEDQLTWTLK	86-104
	Septum formation inhibitor /NP_846895	MEEKKQQNVTIK	1-12
	Hypothetical protein/NP_843342	ELLSENIR	127-134
	Hypothetical protein/ZP_00391958	IEEPENEDKLENK	293-305
	Not characterised	MLAVPHAHQLNILK	
**15**	Amino acid ABC transporter, permease protein/ZP_02216573	ASILSIPKGQWEAAYTIGMTYPQALKR	98-124
	Repeat domain protein/ EJY91466	RLFLSSTEGDADLIGDQALFG*	230-250
	Septum formation inhibitor /NP_846895	MEEKKQQNVTIK	1-12
	Hypothetical protein/ NP_843342	ELLSENIR	127-134
**16**	Amino acid permease/ YP_028011	STELGEQKLNKTK	16-28
	3-hydroxylsobutyrate dehydrogenase /YP_030202.	DGEENSGTQVLYKK	136-149
	2-methylcitrate dehydratase /NP_844732	GWGFQDVLFNKQE*	252-264
	Repeat domain protein/ EJY91466	RLFLSSTEGDADLIGDQALFG*	230-250
	Septum formation inhibitor /NP_846895	MEEKKQQNVTIK	1-12
	Hypothetical protein/NP_847688	MESMIASLEKNDAK	34-47

*: Indicates peptide not cleaved by trypsin

SUPPLEMENTARY DATA 2 ctd.

Identification of peptides in the insoluble fraction (pellet) of the anthrax vaccine

Spot No.	***B. anthracis* protein (% peptide coverage)/NCBI accession number**	***Mr*/*pI* Predicted**	**Peptides identified by LC-MS/MS**
1	Protective antigen(34%) /AAA22637	82.8/5.90	D1 EVISSDNLQLPELK<br>D1 STSAGPTVPDRDNDGIPDSLEVEGYTVDVK<br>D1 WSTASDPYSDFEK<br>D2 AKENQLSQILAPNNYYPSK<br>D2 ENQLSQILAPNNYYPSK<br>D2 LDTDQVYGNIATYNFENGR<br>D2 TWAETMGLNTADTAR<br>D2 TWAETmGLNTADTAR<br>D2-3 VDTGSNWSEVLPQIQETTAR<br>D2 YVNTGTAPIYNVLPTTSLVLGK<br>D3 DLNLVER<br>D3 IAAVNPSDPLETTKPDmTLK<br>D3 NQLAELNATNIYTVLDK<br>D4 EVINSSTEGLLLNIDK<br>D4 NNIAVGADESVVK<br>D4 YDMLNISSLR<br>D4 YDmLNISSLR<br>D4 YNDKLPLYISNPNYK
1	Lethal factor (24.1%)/CAC93932	93.7/5.95	ALNVYYEIGK<br>DSLSEEEKELLNR<br>EGYEPVLVIQSSEDYVENTEK<br>FLDVLNTIK<br>IQEAQLNINQEWNK<br>IQIDSSDFLSTEEK<br>IYIVDGDITK<br>KLQIPIEPK<br>LDIQPYDINQR<br>LKLDIQPYDINQR<br>LLEKVPSDVLEmYK<br>LQDTGGLIDSPSINLDVR<br>LQIPIEPK<br>NASDSDGQDLLFTNQLK<br>TFQFINDQIK<br>TNEAEFFAEAFR<br>VPSDVLEmYK<br>VTNYLVDGNGR

m: oxidized form of methionine, possibly a site of post translational modification

<u>SUPPLEMENTARY DATA: APPENDIX II ctd.</u>

Identification of peptides in the insoluble fraction (pellet) of the anthrax vaccine

Spot No.	*B. anthracis* protein (% peptide coverage)/NCBI accession number	*Mr*/p*I* Predicted	Peptides identified by LC-MS/MS
3	Protective antigen, fragment (15.8%)/AAA22637	82.6/5.72	D1 EVISSDNLQLPELK<br>D1 WSTASDPYSDFEK<br>D2 TWAETMGLNTADTAR<br>D2 TWAETmGLNTADTAR<br>D2 YVNTGTAPIYNVLPTTSLVLGK<br>D3 IAAVNPSDPLETTKPDMTLK<br>D4 LPLYISNPNYK<br>D4 NNIAVGADESVVK<br>D4 VNVYAVTK
3	Lethal factor (6.9%)/CAC93932	93.7/5.95	ALNVYYEIGK<br>FLDVLNTIK<br>IQLSPDTR<br>IYIVDGDITK<br>LQIPIEPK<br>VTNYLVDGNGR
4	Enolase (62.4%)/NP_847538	46.4/4.77	AGENVFLGMDVASSEFYNK<br>AGYTAVVSHR<br>AIVPSGASTGEHEAVELR<br>AMIELDGTPNK<br>AmIELDGTPNK<br>AMIELDGTPNKGK<br>AmIELDGTPNKGK<br>EALEVIIEAIEK<br>GISNSILIK<br>GLNTAVGDEGGFAPNLGSNR<br>GNPTVEVEVYTESGAFGR<br>GVmNAVNNVNEAIAPEIVGFDVTDQAGIDR<br>IEDELGEIAVYDGIK<br>IGDKVQLVGDDLFVTNTQK<br>LGANAILGVSMAVAHAAADFVGLPLYR<br>MGAEVFHALK<br>mGAEVFHALK<br>RAGYTAVVSHR<br>SGETEDATIADIAVATNAGQIK<br>VNQIGTLTETFEAIEMAK<br>VNQIGTLTETFEAIEmAK<br>VQLVGDDLFVTNTQK<br>YDLAGEGR

m: oxidized form of methionine, possibly a site of post translational modification

SUPPLEMENTARY DATA 2 ctd.

Identification of peptides in the insoluble fraction (pellet) of the anthrax vaccine

Spot No.	***B. anthracis* protein (% peptide coverage)/NCBI accession number**	***Mr*/*pI* Predicted**	**Peptides identified by LC-MS/MS**
4	Protective antigen fragment (4.5%)/AAA22637	82.6/5.72	D3 IAAVNPSDPLETTKPDMTLK<br>D4 NNIAVGADESVVK
5	Enolase (58.9%)/NP_847538	46.4/4.77	AGENVFLGmDVASSEFYNK<br>AGYTAVVSHR<br>AMIELDGTPNK<br>AmIELDGTPNK<br>AMIELDGTPNKGK<br>AmIELDGTPNKGK<br>AMIELDGTPNKGKLGANAILGVSMAVAHAAADFVGLPLYR<br>EALEVIIEAIEK<br>ETGKYDLAGEGR<br>GISNSILIK<br>GLNTAVGDEGGFAPNLGSNR<br>GNPTVEVEVYTESGAFGR<br>GVmNAVNNVNEAIAPEIVGFDVTDQAGIDR<br>IEDELGEIAVYDGIK<br>IGDKVQLVGDDLFVTNTQK<br>IGDKVQLVGDDLFVTNTQK<br>MGAEVFHALK<br>mGAEVFHALK<br>SGETEDATIADIAVATNAGQIK<br>VNQIGTLTETFEAIEmAK<br>VQLVGDDLFVTNTQK<br>YDLAGEGR
5	Protective antigen fragment (3.8%)/ AAA22637	82.6/5.72	D2 TWAETmGLNTADTAR<br>D4 NNIAVGADESVVK
6	Lethal factor (12.5%)/CAC93932	93.7/5.95	ALNVYYEIGK<br>FLDVLNTIK<br>IQEAQLNINQEWNK<br>IYIVDGDITK<br>LKLDIQPYDINQR<br>LQDTGGLIDSPSINLDVR<br>NASDSDGQDLLFTNQLK<br>VPSDVLEmYK

m: oxidized form of methionine, possibly a site of post translational modification

SUPPLEMENTARY DATA 2 ctd.

Identification of peptides in the insoluble fraction (pellet) of the anthrax vaccine

Spot No.	***B. anthracis* protein (% peptide coverage)/NCBI accession number**	***Mr*/*pI* Predicted**	**Peptides identified by LC-MS/MS**
6	Protective antigen (34.0%) /AAA22637	82.8/5.90	D1 EVISSDNLQLPELK<br>D2 AKENQLSQILAPNNYYPSK<br>D2 ENQLSQILAPNNYYPSK<br>D2 LDTDQVYGNIATYNFENGR<br>D2 TWAETMGLNTADTAR<br>D2 TWAETmGLNTADTAR2<br>D2-3 VDTGSNWSEVLPQIQETTAR<br>D2-3 VRVDTGSNWSEVLPQIQETTAR<br>D2 YVNTGTAPIYNVLPTTSLVLGK<br>D3 DLNLVER<br>D3 IAAVNPSDPLETTKPDMTLK<br>D3 IAFGFNEPNGNLQYQGK<br>D3 NQLAELNATNIYTVLDK<br>D4 ENTIINPSENGDTSTNGIK<br>D4 EVINSSTEGLLLNIDK<br>D4 FHYDRNNIAVGADESVVK<br>D4 LPLYISNPNYK<br>D4 NNIAVGADESVVK<br>D4 YDMLNISSLR<br>D4 YNDKLPLYISNPNYK
6	Enolase (47.1%) /NP_847538	46.4/4.77	AGENVFLGmDVASSEFYNK<br>AMIELDGTPNK<br>EALEVIIEAIEK<br>GISNSILIK<br>GLNTAVGDEGGFAPNLGSNR<br>GNPTVEVEVYTESGAFGR<br>GVmNAVNNVNEAIAPEIVGFDVTDQAGIDR<br>IEDELGEIAVYDGIK<br>IGDKVQLVGDDLFVTNTQK<br>MGAEVFHALK<br>SGETEDATIADIAVATNAGQIK<br>VNQIGTLTETFEAIEmAK<br>VNQIGTLTETFEAIEMAK<br>VQLVGDDLFVTNTQK
6	Glucose-6-phosphate isomerase (16.4%) /NP_847316	50.3/5.14	AAIEmLNHSFYNTLSK<br>GAAAGRDDFGTSELEENPAYQYAVVR<br>SGTTTEPALAFR<br>TLADNEGYETFVIPDDVGGR

m: oxidized form of methionine, possibly a site of post translational modification

SUPPLEMENTARY DATA 2 ctd.

Identification of peptides in the insoluble fraction (pellet) of the anthrax vaccine

Spot No.	***B. anthracis* protein (% peptide coverage)/NCBI accession number**	***Mr*/*pI* Predicted**	**Peptides identified by LC-MS/MS**
6	Serine hydroxylmethyl transferase (5.3%) /NP_847716	45.1/5.91	LIVAGASAYPR<br>VFAAIEAELGR
7	Protective antigen (31.1%) /AAA22637	82.8/5.90	D1 EVISSDNLQLPELK<br>D2 AKENQLSQILAPNNYYPSK<br>D2 ENQLSQILAPNNYYPSK<br>D2 LDTDQVYGNIATYNFENGR<br>D2 TWAETmGLNTADTAR<br>D2 TWAETMGLNTADTAR<br>D2 YVNTGTAPIYNVLPTTSLVLGK<br>D2-3 VDTGSNWSEVLPQIQETTAR<br>D3 DLNLVER<br>D3 IAAVNPSDPLETTKPDmTLK<br>D3 IAAVNPSDPLETTKPDMTLK<br>D3 IAFGFNEPNGNLQYQGK<br>D4 ENTIINPSENGDTSTNGIK<br>D4 LNAKmNILIR<br>D4 LPLYISNPNYK<br>D4 NNIAVGADESVVK<br>D4 QDGKTFIDFK<br>D4 TFIDFKK<br>D4 VNVYAVTK<br>D4 YNDKLPLYISNPNYK
7	Enolase (35.3%)/NP_847538	46.4/4.77	AMIELDGTPNK<br>AmIELDGTPNK<br>EALEVIIEAIEK<br>GISNSILIK<br>GLNTAVGDEGGFAPNLGSNR<br>GNPTVEVEVYTESGAFGR<br>GVmNAVNNVNEAIAPEIVGFDVTDQAGIDR<br>IEDELGEIAVYDGIK<br>SGETEDATIADIAVATNAGQIK<br>VQLVGDDLFVTNTQK
7	Lethal factor (3.7%)/CAC93932	93.7/5.95	ALNVYYEIGK<br>IYIVDGDITK<br>VPSDVLEmYK

m: oxidized form of methionine, possibly a site of post translational modification

SUPPLEMENTARY DATA 2 ctd.

Identification of peptides in the insoluble fraction (pellet) of the anthrax vaccine

Spot No.	*B. anthracis* protein (% peptide coverage)/NCBI accession number	*Mr*/*pI* Predicted	Peptides identified by LC-MS/MS
7	Glucose-6-phosphate isomerase (4.4%) /NP_847316	50.3/5.14	DLFETVLK<br>SGTTTEPALAFR
8	Protective antigen fragment (21.9%) /AAA22637	62.8/5.83	D2 ENQLSQILAPNNYYPSK<br>D2 TWAETmGLNTADTAR<br>D2 TWAETMGLNTADTAR<br>D3 DLNLVER<br>D3 IAAVNPSDPLETTKPDmTLK<br>D4 ENTIINPSENGDTSTNGIK<br>D4 LPLYISNPNYK<br>D4 NNIAVGADESVVK<br>D4 TFIDFKK<br>D4 YDmLNISSLR<br>D4 YDMLNISSLR<br>D4 YNDKLPLYISNPNYK
9	Protective antigen fragment (45.1%) /AAA22637	62.8/5.83	D2 AKENQLSQILAPNNYYPSK<br>D2 ENQLSQILAPNNYYPSK<br>D2 LDTDQVYGNIATYNFENGR<br>D2 NLAPIALNAQDDFSSTPITmNYNQFLELEK<br>D2 TWAETMGLNTADTAR<br>D2-3 VDTGSNWSEVLPQIQETTAR<br>D2-3 VRVDTGSNWSEVLPQIQETTAR<br>D2 YVNTGTAPIYNVLPTTSLVLGK<br>D3 DLNLVER<br>D3 IAAVNPSDPLETTKPDmTLK<br>D3 IAAVNPSDPLETTKPDMTLK<br>D3 IAFGFNEPNGNLQYQGK<br>D3 NQLAELNATNIYTVLDK<br>D3 RIAAVNPSDPLETTKPDmTLK<br>D4 ENTIINPSENGDTSTNGIK<br>D4 LPLYISNPNYK<br>D4 NNIAVGADESVVK<br>D4 TFIDFKK<br>D4 YDmLNISSLR<br>D4 YDMLNISSLR<br>D4 YNDKLPLYISNPNYK

m: oxidized form of methionine, possibly a site of post translational modification

SUPPLEMENTARY DATA 2 ctd.

Identification of peptides in the insoluble fraction (pellet) of the anthrax vaccine

Spot No.	*B. anthracis* protein (% peptide coverage)/NCBI accession number	*Mr*/p*I* Predicted	Peptides identified by LC-MS/MS
11	Protective antigen fragment (18.9%) /AAA22637	26.6/7.46	D1 EVISSDNLQLPELK<br>D1 STSAGPTVPDRDNDGIPDSLEVEGYTVDVK
11	Lipo protein, Bmp family (6.5%) /NP_846172	38.3/8.48	DADYIPNLTK<br>VGMVTDVGGVDDK
11	6-Phosphofructo kinase (8.5%) /NP_847047	34.3/6.29	IGVLTSGGDSPGmNAAIR<br>TYVIEVmGR
12	Protective antigen fragment (23.2%) /AAA22637	26.6/7.46	D1 EVISSDNLQLPELK<br>D1 STSAGPTVPDRDNDGIPDSLEVEGYTVDVK<br>D1 IDKNVSPEAR
12	Iron compound ABC transporter, iron compound-binding protein (21.0%) /NP_847506	35.9/6.71	LVPNAAVIDLNVDVSEK<br>NVVALDNR<br>PALQNTTAVSK<br>QLVADFDKSIEK<br>SPALQNTTAVSKK<br>VmSVIVTGGNIGFAAPHSGR
12	Lipoprotein, Bmp family (6.5%) /NP_846172	38.3/8.48	DADYIPNLTK<br>VGmVTDVGGVDDK
13	Protective antigen fragment (16.5%) /AAA22637	82.6/5.72	D1 EVISSDNLQLPELK<br>D1-2 HPLVAAYPIVHVDMENIILSK<br>D1-2 HPLVAAYPIVHVDmENIILSK<br>D1-2 HPLVAAYPIVHVDMENIILSKNEDQSTQNTDSQTR<br>D1 KEVISSDNLQLPELK<br>D1 LYWTDSQNK<br>D1 RTFLSPWISNIHEK<br>D1 STSAGPTVPDRDNDGIPDSLEVEGYTVDVK<br>D1 SSPEKWSTASDPYSDFEK<br>D1 WSTASDPYSDFEK
13	Iron compound ABC transporter, iron compound-binding protein (11.7%) /NP_847506	35.9/6.71	TFETLADWGIK<br>LVPNAAVIDLNVDVSEK<br>GLKDSTITLGK
13	Lethal factor (2.7%)/CAC93932	93.7/5.95	TNEAEFFAEAFR<br>TFQFINDQIK

m: oxidized form of methionine, possibly a site of post translational modification

SUPPLEMENTARY DATA 2 ctd.

Identification of peptides in the insoluble fraction (pellet) of the anthrax vaccine

Spot No.	*B. anthracis* protein (% peptide coverage)/NCBI accession number	*Mr*/p*I* Predicted	Peptides identified by LC-MS/MS
13	6-Phosphofructo kinase (9.7%) /NP_847047	34.3/6.29	LVDHDIIEALAQK<br>AVELLIAGK<br>TYVIEVmGR
13	Lipoprotein, Bmp family (6.5%) /NP_846172	38.3/8.48	VGMVTDVGGVDDK<br>DADYIPNLTK
13	Edema factor fragment (5.2%) /AAA79215	29.4/5.19	DKSLDPEFLNLIK<br>SLDPEFLNLIK
14	Lethal factor (12.5%)/CAC93932	93.7/5.95	GLYVPESR<br>IQEAQLNINQEWNK<br>IQLSPDTR<br>LDIQPYDINQR<br>LQDTGGLIDSPSINLDVR<br>SILLHGPSK<br>TFQFINDQIK<br>TNEAEFFAEAFR<br>VTNYLVDGNGR
16	Protective antigen fragment (21.0%) /AAA22637	82.6/5.72	D1 EVISSDNLQLPELK<br>D1 KEVISSDNLQLPELK<br>D1 RSTSAGPTVPDRDNDGIPDSLEVEGYTVDVK<br>D1 SDEYTFATSADNHVTmWVDDQEVINK<br>D1 STSAGPTVPDRDNDGIPDSLEVEGYTVDVK<br>D3 IAAVNPSDPLETTKPDmTLK<br>D3 IAFGFNEPNGNLQYQGK<br>D3 NQLAELNATNIYTVLDK<br>D4 LPLYISNPNYK<br>D4 NNIAVGADESVVK<br>D4 YNDKLPLYISNPNYK
16	Lethal factor (8.2%)/CAC93932	93.7/5.95	ALNVYYEIGK<br>FLDVLNTIK<br>IYIVDGDITK<br>NASDSDGQDLLFTNQLK<br>TFQFINDQIK<br>VPSDVLEmYK
16	S-layer protein (4.3%)/ NP_052786	76.2/9.16	EQVAAVIYR<br>FGLGDSVTR<br>NAFNIPVVGK

m: oxidized form of methionine, possibly a site of post translational modification

SUPPLEMENTARY DATA 2 ctd.

Identification of peptides in the insoluble fraction (pellet) of the anthrax vaccine

Spot No.	*B. anthracis* protein (% peptide coverage)/NCBI accession number	*Mr*/*pI* Predicted	Peptides identified by LC-MS/MS
16	DNA-binding response regulator (12.9%) /NP_845926	25.8/5.17	EIVSDYFR<br>ESAVPIIMLTAR<br>ESAVPIImLTAR<br>TVLLVEDER
16	Formate acetyl-transferase (6.1%) /NP_843045	84.5/5.96	KADLNLTGGVmSEDTmR<br>KSGVITGLPDAYGR<br>SGVITGLPDAYGR<br>THNQGVFDAYTPEmR
16	6-Phosphofructo kinase (11.3%) /NP_847047	34.3/6.29	TYVIEVmGR<br>IGVLTSGGDSPGmNAAIR<br>AVELLIAGK
16	Sporulation-control protein Spo0M (13.5%)/NP_844693	28.8/4.94	ITTTTYDLER<br>FTVTNEDIPTMK<br>MPVETPLTLGmK<br>mPVETPLTLGmK
16	Transaldolase (20.3%)/NP_845714	24.0/5.47	AGATYVSPFLGR<br>LGVLAGVTTNPSLVAK<br>VIEQLAmHPLTDQGIEK
16	Purine nucleoside phosphorylase DeoD-type (9.4%)/ NP_843936	25.7/5.19	GmLGFTGTYK<br>VGNVLTADVFYR
16	2,3,4,5 -tetrahydro pyridine 2,6-di-carboxyl ate N-succinyl transferase (10.4%) /NP_846430	25.7/5.54	mKmmDANEIISFIQK<br>NSAIPmLDLK
18	Manganese dependent superoxide dismutase fragment (21.1%) /NP_846724	15.2/5.41	SVEELVANLNEVPEAIR
18	S-layer protein (2.9%) / NP_052786	76.2/9.16	EQVAAVIYR<br>NAFNIPVVGK
19	Protective antigen fragment (6.4%) /AAA22637	26.6/7.46	D1 EVISSDNLQLPELK<br>D1 KEVISSDNLQLPELK
20	Protective antigen fragment (10.3%) /AAA22637	26.6/7.46	D1 EVISSDNLQLPELK<br>D1 KEVISSDNLQLPELK<br>D1 LYWTDSQNK

m: oxidized form of methionine, possibly a site of post translational modification

SUPPLEMENTARY DATA 2 ctd.

Identification of peptides in the insoluble fraction (pellet) of the anthrax vaccine

Spot No.	*B. anthracis* protein (% peptide coverage)/NCBI accession number	*Mr*/*pI* Predicted	Peptides identified by LC-MS/MS
20	S-layer protein (3.7%)/ NP_052786	76.2/9.16	KQQELESELVQLNK<br>QQELESELVQLNK<br>SGQYLYYINK
20	Hypothetical protein (6.4%) /NP_843713	19.9/7.31	LGIVIFPSK<br>mKLGIVIFPSK
20	Glucose-6-phosphate isomerase (6.2%) /NP_847316	50.3/5.14	AAIEmLNHSFYNTLSK<br>SGTTTEPALAFR
20	Lethal factor fragment (7.6%) /CAC93932	30.3/5.14	ALNVYYEIGK<br>IYIVDGDITK
23	Nucleoside diphosphate kinase (39.9%) /NP_843987	16.5/5.55	AFIGEIVAR<br>EIGIFFKEEELVDYSK<br>GFQLVGAK<br>NIIHGSDSLESAER<br>TFLmVKPDGVQR
24	Iron transport-associated protein (26.1%) /YP_006211623	98.9/5.62	ALKDQTDEISMmNTYTK<br>DGEYSIGFK<br>DGEYSINFK<br>DKTEEISMmNQYVVSPAR<br>DQTDEISmMNTYTK<br>LADGEYSIGFK<br>LVATNVISEDK<br>mNTYmVNPGVLK<br>MNTYmVNPGVLK<br>NFQTEKDGAFVDAK<br>NLKDGQYDIAFK<br>NQTDEESKmNTYmVNPGVLK<br>TEEISmMNTYTK<br>VFIEMASR<br>VLKDKTEEISmmNQYVVSPAR<br>VLKDTSDEESmMNQYSVSPGTLK<br>VLKDTSDEESMmNQYSVSPGTLK<br>VVEFDVEDVEK<br>VVEFEANDLFAK<br>VVEFEVADLSK<br>VVEVAVDDLSK<br>VVEVEVADLSK<br>YIAMTLK

m: oxidized form of methionine, possibly a site of post translational modification

SUPPLEMENTARY DATA 2 ctd.

Identification of peptides in the insoluble fraction (pellet) of the anthrax vaccine

Spot No.	*B. anthracis* protein (% peptide coverage)/NCBI accession number	*Mr*/p*I* Predicted	Peptides identified by LC-MS/MS
25	Protective antigen (18.9%) /AAA22637	82.8/5.90	D1 EVISSDNLQLPELK<br>D1 STSAGPTVPDRDNDGIPDSLEVEGYTVDVK<br>D2 TWAETmGLNTADTAR<br>D2 TWAETMGLNTADTAR<br>D3 DLNLVER<br>D3 IAAVNPSDPLETTKPDmTLK<br>D4 ENTIINPSENGDTSTNGIK<br>D4 LPLYISNPNYK<br>D4 NNIAVGADESVVK<br>D4 YDmLNISSLR
25	Lethal factor (14.8%)/CAC93932	93.7/5.95	ALNVYYEIGK<br>FLDVLNTIK<br>IQEAQLNINQEWNK<br>IYIVDGDITK<br>LQIPIEPK<br>NASDSDGQDLLFTNQLK<br>NNIQSDLIK<br>TFQFINDQIK<br>TNEAEFFAEAFR<br>VPSDVLEmYK<br>VPSDVLEMYK<br>VTNYLVDGNGR
25	S-layer protein SAP (2.6%) /NP_843397	86.5/7.30	TEVAVGSLEAK<br>TVEIEAFAQK
25	Edema factor (3.7%) /AAA79215	88.8/6.89	INTIPTSAEFIK<br>SLSDDSDSSDLLFSQK

m: oxidized form of methionine, possibly a site of post translational modification
